# Hydroxyapatite-binding Silver/Titanium Dioxide as a Potential Control Compound Against Mosquito Vectors, *Aedes aegypti* (Diptera: Culicidae) and *Anopheles dirus* (Diptera: Culicidae)

**DOI:** 10.1093/jme/tjac175

**Published:** 2022-11-14

**Authors:** Raweewan Srisawat, Patchara Sriwichai, Jiraporn Ruangsittichai, Chawarat Rotejanaprasert, Naoko Imaizumi, Dai Yamaki, Maki Maekawa, Yuki Eshita, Narumi Okazaki

**Affiliations:** Department of Medical Entomology, Faculty of Tropical Medicine, Mahidol University, Ratchathewi, Bangkok 10400, Thailand; Department of Medical Entomology, Faculty of Tropical Medicine, Mahidol University, Ratchathewi, Bangkok 10400, Thailand; Department of Medical Entomology, Faculty of Tropical Medicine, Mahidol University, Ratchathewi, Bangkok 10400, Thailand; Department of Tropical Hygiene, Faculty of Tropical Medicine, Mahidol University, Ratchathewi, Bangkok 10400, Thailand; Mahidol-Oxford Tropical Medicine Research Unit, Faculty of Tropical Medicine, Mahidol University, Ratchathewi, Bangkok 10400, Thailand; DR.C Medical Medicine Co., Ltd., Shinjuku, Tokyo 160-0023, Japan; DR.C Medical Medicine Co., Ltd., Shinjuku, Tokyo 160-0023, Japan; Seltec Co., Ltd., Hachioji, Tokyo 192-0062, Japan; International Institute for Zoonosis Control, Hokkaido University, Sapporo, Hokkaido 001-0020, Japan; DR.C Medical Medicine Co., Ltd., Shinjuku, Tokyo 160-0023, Japan

**Keywords:** vector control, hydroxyapatite-binding silver/titanium dioxide, mortality, submersion rate, hatching rate

## Abstract

Controlling mosquitoes is vital for counteracting the rising number of mosquito-borne illnesses. Vector control requires the implementation of various measures; however, current methods lack complete effectiveness, and new control agents or substances are urgently needed. Therefore, this study developed a nonwoven fabric sheet coated with hydroxyapatite-binding silver/titanium dioxide compound (hydroxyapatite-binding silver/titanium dioxide sheet [HATS])and evaluated its effectiveness on all stages of laboratory *Aedes aegypti* (Linnaeus); Diptera: Culicidae and *Anopheles dirus* (Peyton & Harrison); Diptera: Culicidae. We reared larvae with HATS and control sheets and assessed their mortality, emergence, and hatching rates. The submersion rates of engorged female mosquitoes in submerged HATS and control sheets were also compared. The HATS strongly affected mosquito development, resulting in high mortality rates (mean ± SE) of 99.66 ± 0.58% (L1–L2) and 91.11 ± 9.20% (L3–L4) for *Ae. aegypti* and 100% of both stages for *An. dirus*. In contrast, mosquitoes raised in the control sheet showed relatively high survival rates of 92.33 ± 3.21% (L1–L2) and 95.67 ± 0.58% (L3–L4) for *Ae. aegypti* and 86.07 ± 3.53% (L1–L2) and 92.01 ± 8.67% (L3–L4) for *An. dirus.* Submersion of engorged females was found in the HATS oviposition cup, leading to a decreased number of eggs and a low hatching rate compared to that of the control. Overall, HATS may be a useful new control method for *Ae. aegypti* and *An. dirus*.

## Graphical Abstract

**Graphical Abstract:**
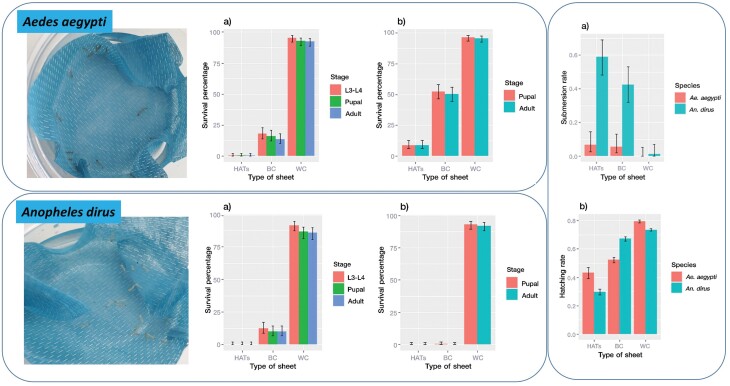

The mosquito is one of the most dangerous insects in the world (Daniel and Wunderman 2020). Infected mosquitoes can carry and spread diseases, such as dengue fever and malaria to humans through bites. Dengue fever has recently become more widespread globally (WHO 2021). Currently, approximately half of the human global population has a risk of contracting dengue. The first outbreak of dengue hemorrhagic fever occurred in Thailand in 1958 (Rojanapithayakorn 1998), and its prevalence has undergone a cyclic increase. The principal dengue vector in Thailand, *Aedes aegypti* (Linnaeus; Diptera: Culicidae), was originally reported in 1907 (Theobald) and is also an arbovirus vector of Zika yellow fever, and chikungunya virus (WHO 2021). It is a daytime biting mosquito, with peak bite periods in the early morning and before dusk, and a multiple feeder, allowing it to spread mosquito-borne diseases more efficiently (Trpis et al. 1973). The eggs laid by a female can persist for several months before hatching, which occurs during contact with water. 

One of Thailand’s major malaria vectors is *Anopheles dirus* (Peyton & Harrison); Diptera: Culicidae, which is found all over Thailand. It feeds between sunset and midnight, a behavior known as exophagic and anthrophilic (Tainchum et al. 2015, Tananchai et al. 2019). Owing to the increasing number of mosquito-borne illnesses and the lack of a completely effective vaccine, the prevention of these diseases is primarily achieved through effective mosquito control (WHO 2009, Amarasinghe et al. 2020).

On the one hand, chemical control using insecticides has been successful in controlling the target vector species, such as *Aedes* (Srisawat et al. 2010) and *Anopheles* (Corbel et al. 2013). On the other hand, mosquitoes are increasingly becoming less susceptible and developing resistance to chemical insecticides (Hemingway et al. 2004, Dusfour et al. 2019, Duarte et al. 2020), resulting in a rebound in vectorial capacity (Elumalai et al. 2017). Therefore, it is required to identify an agent that reduces the selection pressure of insecticide resistance and is easily degradable, safe, and environmentally friendly (Manjarres-Suarez and Olivero-Verbel 2013). Recently, metal nanoparticles have received substantial attention owing to their desirable characteristics, such as inexpensiveness, having a wide range of applications, excellent antimicrobial activity, less toxicity to humans, ecofriendliness (Santhosh et al. 2015, Kumar et al. 2020, Narayanan et al. 2021), and larvicidal effect against mosquitoes (Patil et al. 2012, Kumar et al. 2017, Pilaquinga et al. 2019). Silver is the most popular metal for the synthesis of nanoparticles (AgNPs) from plant extracts for use in insect vector control, and those generated from *Avicennia marina* (Barnawi et al. 2019), *Annona squamosa* (Arjunan et al. 2012), *Lippia citriodora* (Elemike et al. 2017), *Derris trifoliata* (Kumar et al. 2017), *Leucas aspera*, and *Hyptis suaveolens* (Elumalai et al. 2017) exhibit mosquitocidal effect against *Ae. aegypti*, *An. stephensi* Liston (Diptera: Culicidae), and *Culex quinquefasciatus* Say (Diptera: Culicidae).

The fabrication of titanium dioxide (TiO_2_) has received increased attention recently owing to its versatile applications, such as in healthcare products (sunscreen lotions, beauty creams, skin ointments, etc.) and electronic industries (capacitors, electrochemical electrodes, solar cells, etc.) (Kamaraj et al. 2010). The TiO_2_NPs prepared from the plant extracts of *Pouteria campechiana* (Narayanan et al. 2021), *Parthenium hysterophorus* (Thandapani et al. 2018), *Ficus religiosa* (Soni and Dhiman 2020), and *Morinda citrifolia* (Suman et al. 2015) exhibited larvicidal effects against mosquitoes. These plant extracts reduced and stabilized titanium as nanoparticles. Green TiO_2_NPs have a higher toxicity than plant extracts alone.

A hydroxyapatite-binding silver/titanium dioxide compound (produced by DR.C Medical Medicine Co., Ltd.) has been proposed as a new mosquito control chemical (DR.C Medical Medicine Co., Ltd. n.d.-a). It was first developed as an innovative treatment for sheets and masks that protect against pollen allergies and was later used by several Japanese companies to manufacture items, such as shirts, socks, and towels, that prevent and delay bacterial growth (DR.C Medical Medicine Co., Ltd. n.d.-b). Although the apatite-TiO2-coated cotton fabric can decompose antigenic proteins, bacteria, pollen, mold, and viruses (Okazaki et al. 2022), its ability to kill mosquitoes has not yet been investigated. Only the application of AgNPs coated with natural active ingredients that kill mosquitoes has been reported. There have been several reports of using AgNPs to kill mosquitoes at all life stages. Balakrishnan et al. (2016) used positively synthesized AgNPs from mangrove plants as a larvicide against *Ae. aegypti* and *An. stephensi*, and Shanmugam et al. (2014) assessed the effectiveness of positively green-synthesized AgNPs as a larvicide and pupacide against these two mosquito species. Furthermore, Murugan et al. (2016) reported the use of synthesized TiO_2_NPs as a larvicide and pupacide against *Ae. aegypti*.

The compound is a catalytic substance of anatase-type titanium oxide with added silver that uses hydroxyapatite. The titanium dioxide is a photocatalytic substance that can break down organic compounds, microbial organisms, such as viruses and bacteria, and cancer cells. It has been used for the sterilization of medical devices, food preparation surfaces, air conditioning filters, and sanitary-ware surfaces (Okazaki and Hoshi 2016). To functionalize the compound, TiO_2_ is irradiated with energy greater than its band gap energy, and an electron is excited from the valence band to the conduction band. Therefore, electron-hole pairs are formed that react with water or oxygen molecules to form various reactive oxygen species (ROS). The titanium dioxide with additional silver exhibits efficient photocatalytic properties and antibacterial activity. Hydroxyapatite absorbs bacteria, microbes, pollen, mold, proteins, and viruses, which are then broken down by free radicals of the TiO_2_ photocatalyst (Taoda 2009).

In preliminary studies, the compound was able to kill mosquito larvae and is, therefore, a promising larvicidal agent. However, its safety is an important concern. The HATS has been registered with the Pharmaceuticals and Medical Devices Agency (PMDA, https://www.pmda.go.jp/english/index.html) in Japan and was confirmed to be a safe product for humans and animals (Okazaki and Hoshi 2016), which are vital properties for a vector control agent. Herein, we evaluated the effectiveness of HATS against mosquitoes.

## Materials and Methods

### HATS

**Fig. 1.**
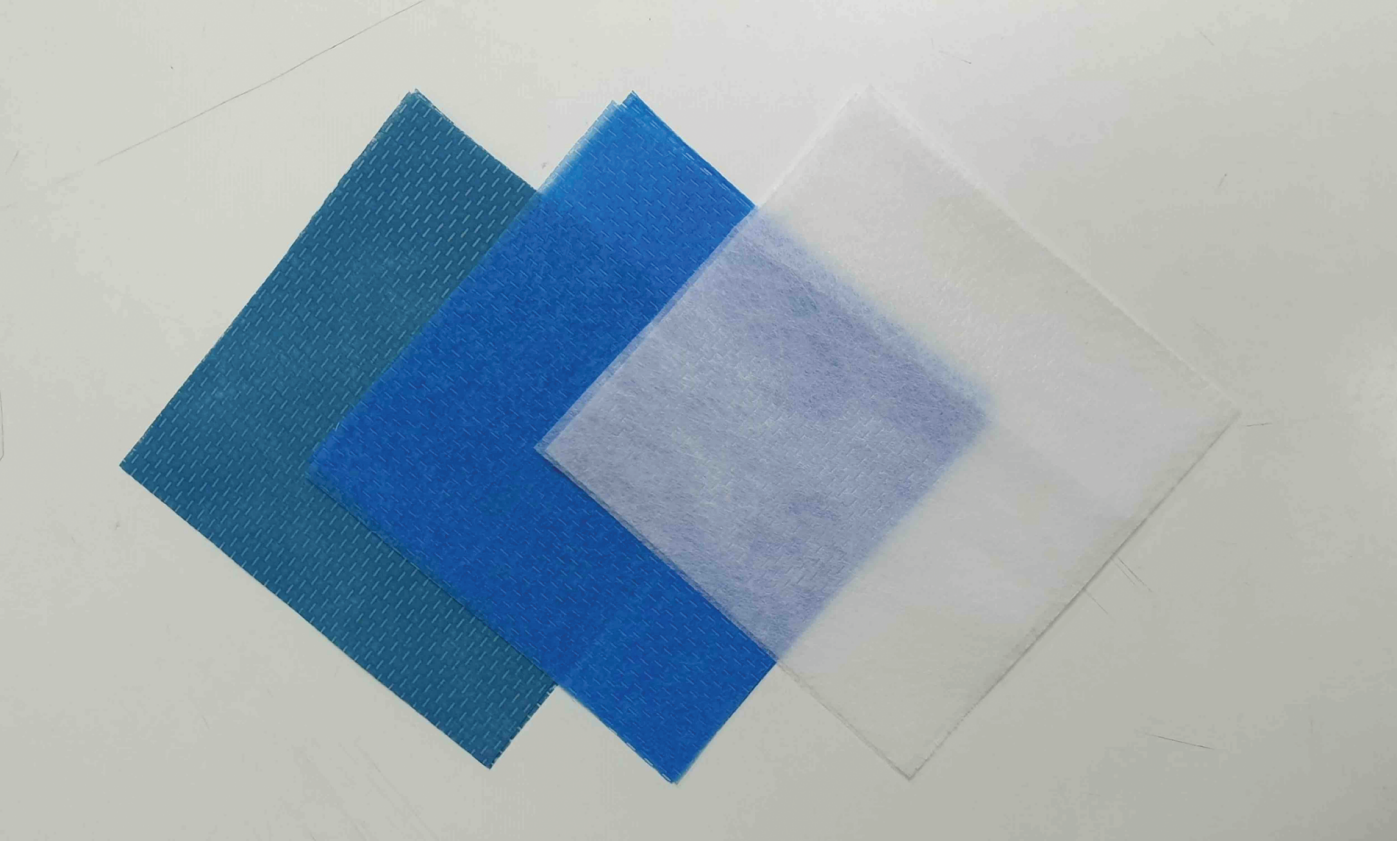
Three types of sheets used in this study. Left – a non-woven fabric sheet of 1 × 1 m containing 13.5 g of hydroxyapatite-binding silver/titanium dioxide compound, defoamer, and phthalocyanine blue (HATS). Middle – a nonwoven fabric containing only defoamer and phthalocyanine blue (BC). Right – nonwoven fabric sheet (WC). Abbreviations: HATS, hydroxyapatite-binding silver/titanium dioxide sheet; BC, blue sheet control; WC, white sheet control.

A dark blue nonwoven fabric sheet of 1 × 1 m (DR.C Medical Medicine Co., Ltd.) containing 13.5 g of HATS, defoamer, and phthalocyanine blue color was used as the experimental sheet (HATS). Two control sheets were used: a light blue nonwoven fabric containing only a defoamer and phthalocyanine blue coloring (original color before compound treatment; blue sheet control, BC) and a non-woven fabric sheet of white color (white sheet control, WC) (Fig. 1).

### Mosquitoes

Laboratory colonies of *Ae. aegypti* (Bora Bora strain) and *An. dirus* (Khao Mai Kaew strain) were used in this study. To maintain mosquitoes in the laboratory, eggs were hatched in plastic trays (20 × 30 × 5 cm). After hatching, 200 larvae were reared in plastic trays with 1,500 ml of dechlorinated water and fed fish food powder daily (Optimum Hi Pro Growth and Color, Perfect Companion Group, Bangkok, Thailand). Pupae were transferred to plastic cups and placed in a cage (20 × 20 × 30 cm) until emergence. Adult mosquitoes were fed 5% sugar solution on cotton wool, which was changed weekly. They were reared and maintained at 25 ± 2°C and 65 ± 10% relative humidity at the insectarium in the Department of Medical Entomology, Faculty of Tropical Medicine, Mahidol University.

#### Early-stage Mosquito Larvae

Mosquito eggs were immersed in a 250-mL cup of reverse osmosis (RO) water (100 mL) lined with a HATS, BC, or WC sheet for one to two days. The hatched larvae (L1-L2) were used as early-stage larvae in this study.

#### Late-stage Mosquito Larvae

Eggs hatched in 100 ml RO water were allowed to develop into L3–L4 larvae for use in the late-stage study. The larvae were fed daily with fish food (Optimum Hi Pro Growth and Color) and maintained at 25 ± 2°C until they reached L3–L4 stage.

### Effect of HATS on the Early- (L1–L2) and Late-stage (L3–L4) of *Ae. aegypti* and *An. dirus* Larvae

RO water (100 ml) was added to a 250-ml plastic cup (9.2 × 7.6 × 5.5 cm top diameter, bottom diameter, and height, respectively, #PC250 MB, FP Chupa Corporation, Tokyo, Japan) with a 2 cm high water column, 56.72 cm^2^ surface area, and 45.34 cm^2^ bottom surface area. Sections (11 × 11 cm) of HATS, BC, and WC sheets were submerged in the bottom of separate cups. There was 0.1635 g of hydroxyapatite-binding silver/titanium dioxide compound particles (=0.11 × 0.11 m × 13.5 g/m^2^). For each mosquito stage, 20 larvae were added to each experimental and control cup, with five cups per treatment. The entire experiment was replicated three times. The larvae were reared at 25 ± 2°C with 0.0046 ± 0.0008 g of larval food provided daily (fish food: Optimum Hi Pro Growth and Color) until they developed into adults. The daily number of larvae, pupae, and adults in each cup was recorded, and the survival rate was calculated using the following formula:


Survival rate = (number of live larvae, pupae, or adult/number of larvae tested) × 100

### Effect of HATS on Submersion of Female Mosquitoes and Egg Hatching for *Ae. aegypti* and *An. dirus*

#### Oviposition Cup

For *An. dirus*, three 250-ml plastic oviposition cups were used containing 80 ml of RO water and four pieces (5.5 × 5.5 cm) of the sheets (HATS, BC, WC) submerged in the bottom of each cup. For *Ae. aegypti*, one (11 × 11 cm) and two pieces (5.5 × 5.5 cm) of each sheet were submerged in three oviposition cups. The number of sheets was verified from our preliminary tests (unpublished data)—to determine the number of HATS that affect mosquito egg hatching rates and adult submergence rates. The tests suggested that *Ae. aegypti* required approximately 1.5 times the number of HATS.

#### Submersion of Female Mosquitoes

Thirty fully blood-fed inseminated female mosquitoes, at 4 d post-blood feeding, were released into each cage. The individual oviposition cups were transferred to each adult mosquito cage, and three cages were used for each species. The female mosquitoes were maintained for 3 d. The total number of females that submerged and laid eggs was recorded after three days of exposure. Three replicates were performed.

#### Egg Hatching

*An. dirus* eggs laid on the sheet were maintained in the same cup until they hatched. *Ae. aegypti* eggs were dried at 27–29°C for 3 d and then submerged in the cup. The number of hatched larvae was counted for one week.

### Data Analysis

Continuous data from each experiment were tested for normal distribution. The non-normally distributed data were analyzed using non-parametric tests. We evaluated the differences among the sheets using the Kruskal–Wallis test of one way analysis of variance (Kruskal and Wallis 1952). For the survival rate and submersion proportions, the multi-sample test for equality of proportions with chi-square test was applied, followed by pairwise comparison using Bonferroni correction. Statistical significance was set at *p* < 0.05 and analyzed using PASW for Windows version 18.0 and R version 1.4.1717. The proportions were calculated using the following formulas:


Egg hatching rate = (number of larvae hatched/number of eggs oviposited) × 100

Submersion proportion of adult mosquitoes = total number of adult mosquitoes submerged/number of adults tested


Survival rate = (number of live larvae, pupae, or adult/number of larvae tested) × 100

## Results

### Effect of HATS on Early- (L1–L2) and Late-stage (L3–L4) Mosquito Larvae

#### Aedes aegypti

**Fig. 2.**
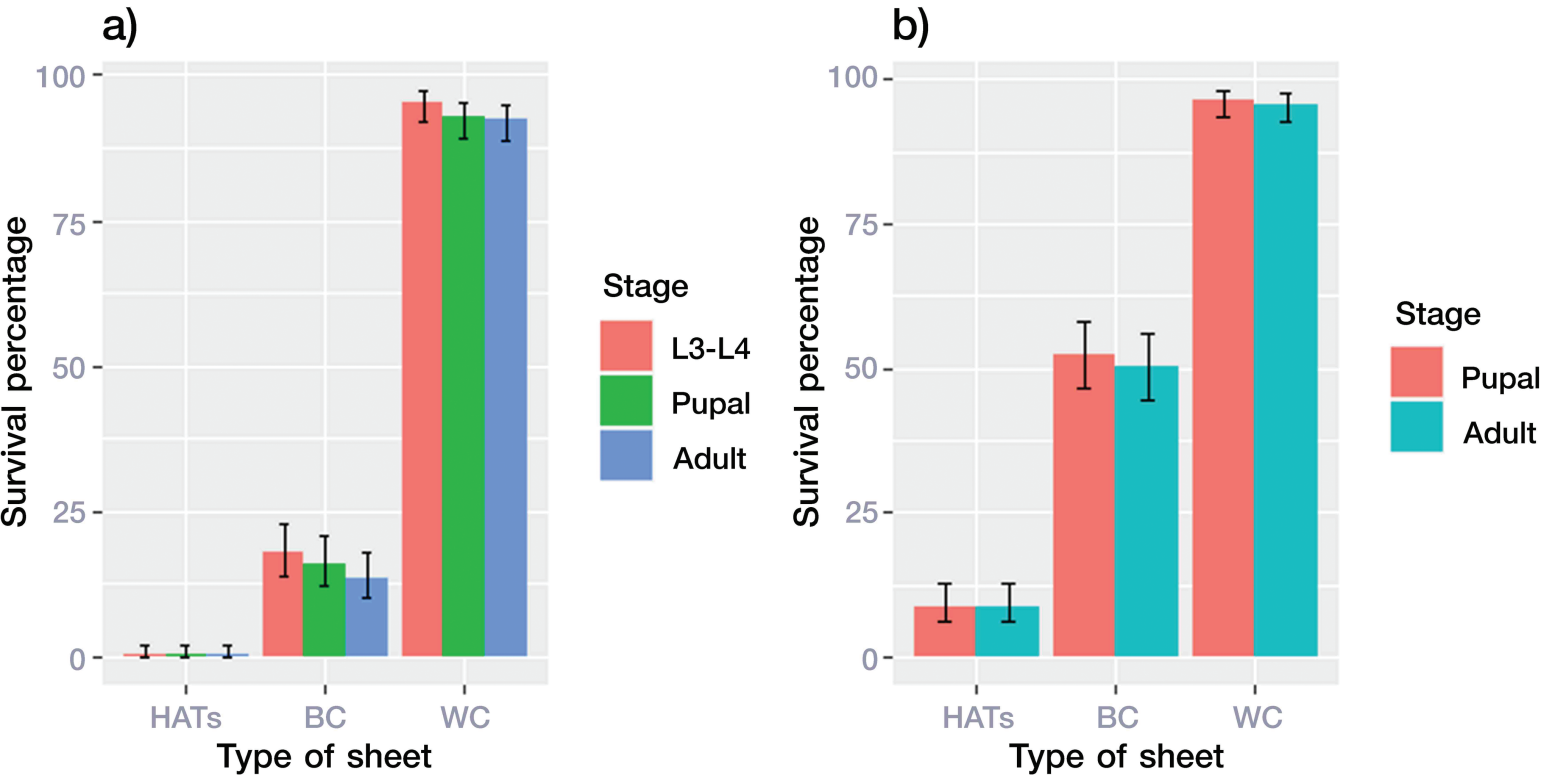
Survival of *Ae. aegypti* after exposure to HATS, BC, or WC. a) Survival ratios of L3–L4, pupal, and adult stages after exposure to the sheets as early-stage larvae (L1–L2). b) Survival ratios of pupal and adult stages after exposure to the sheets as late-stage larvae (L3–L4). The error bars indicate the standard error. Abbreviations: Refer to Fig. 1 on HATS, BC, and WC; RO, reverse osmosis water.

When early-stage larvae (L1-L2) of *Ae. aegypti* were stored in containers with HATS, BC, and WC sheets, there were significant differences in the survival proportions among sheet types, with the highest in WC and the lowest in HATS containers (Fig. 2a) (Kruskal-Wallis, *H* = 40.232, df = 2, *p* < 0.0001). The survival proportion of larvae from the HATS group, i.e., the percentage that successfully developed to the adult stage, was only 0.34%, whereas that of the L3–L4 larvae, pupae, and adults gradually decreased to 18%, 16%, and 14%, respectively, in the BC group. Most early-stage larvae (L1–L2) in the WC container developed into adults (93%) (Fig. 2a).

When the late-stage larvae (L3–L4) of *Ae. aegypti* were maintained in a HATS, BC, or WC sheet container, the final survival proportions in HATS (9% at pupal stage, 9% at adult stage) were significantly different from those in the WC (96% at pupal stage, 96% at adult stage) and BC (52% at pupal stage, 50% at adult stage) containers (Fig. 2b) (Kruskal–Wallis, *H* = 39.024, df = 2, *p* < 0.0001). Early-stage *Ae. aegypti* larvae were more sensitive to HATS than late-stage *Ae. aegypti* larvae, and HATS had a strong lethal effect on *Ae. aegypti* of both stages.

#### Anopheles dirus

**Fig. 3.**
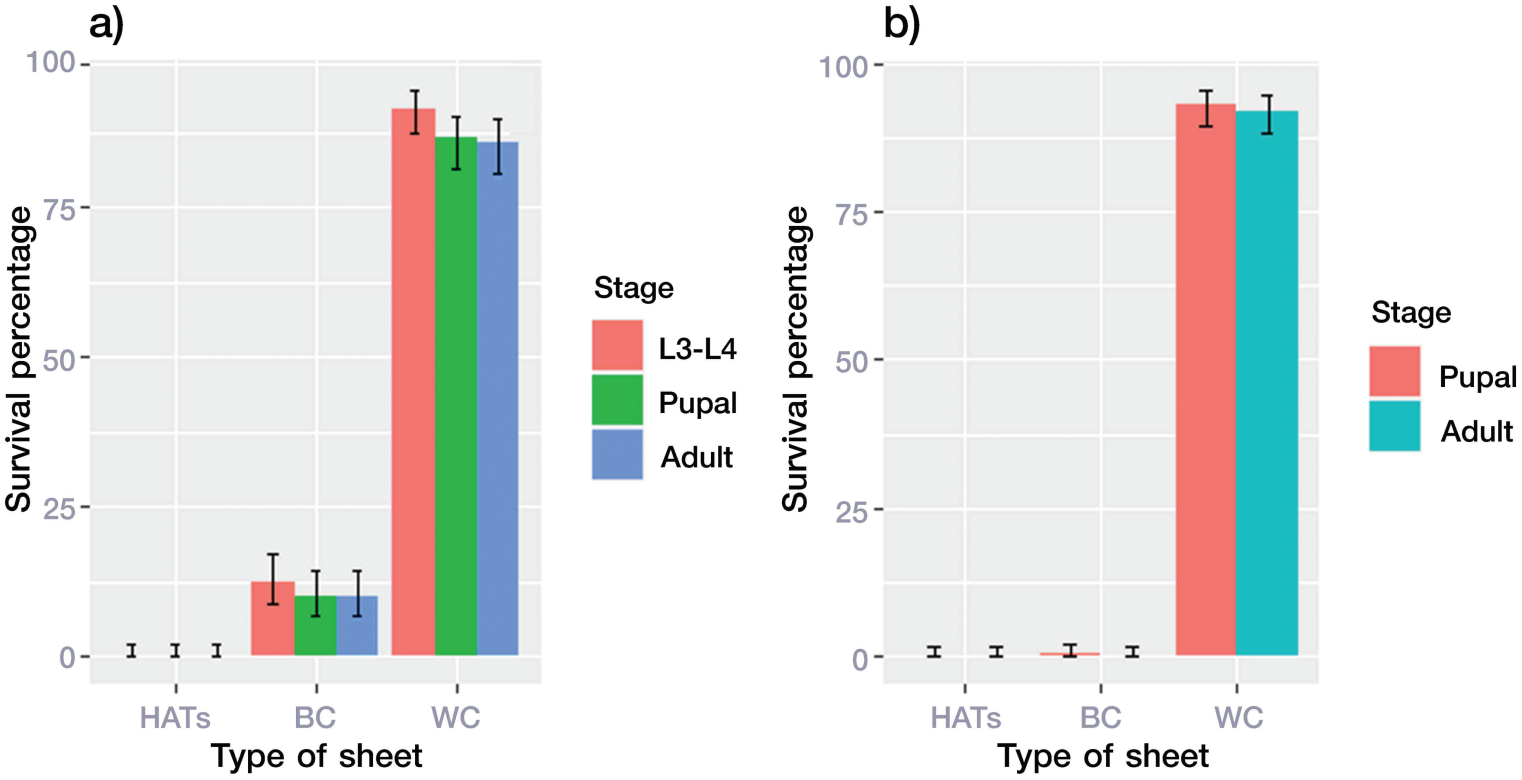
Survival of *An. dirus* after exposure to HATS, BC, or WC. a) Survival ratios of L3–L4, pupal, or adult stages after exposure to the sheets as early-stage larvae (L1–L2). b) Survival ratios of pupal and adult stages after exposure to the sheets as late-stage larvae (L3–L4). The error bars indicate the standard error. Abbreviations: Refer to Fig. 1 on HATS, BC, and WC; RO, reverse osmosis water.

No surviving pupal or adult *An. dirus* were observed in the HATS containers of early-stage (L1–L2) or late-stage (L3–L4) larvae. In the BC sheet containers, some early-stage larvae developed into pupae (14%) and adults (14%) (Fig. 3a), but almost none of the late-stage larvae developed further (Fig. 3b). It was more difficult for the late-stage *An. dirus* living in HATS and BC sheet containers to develop into pupae and adults than for the early-stage larvae. However, the survival percentages in HATS and BC were different from those in the WC (Kruskal–Wallis, *H* = 41.862, df = 2, *p* < 0.0001).

### Developing Time

**Fig. 4.**
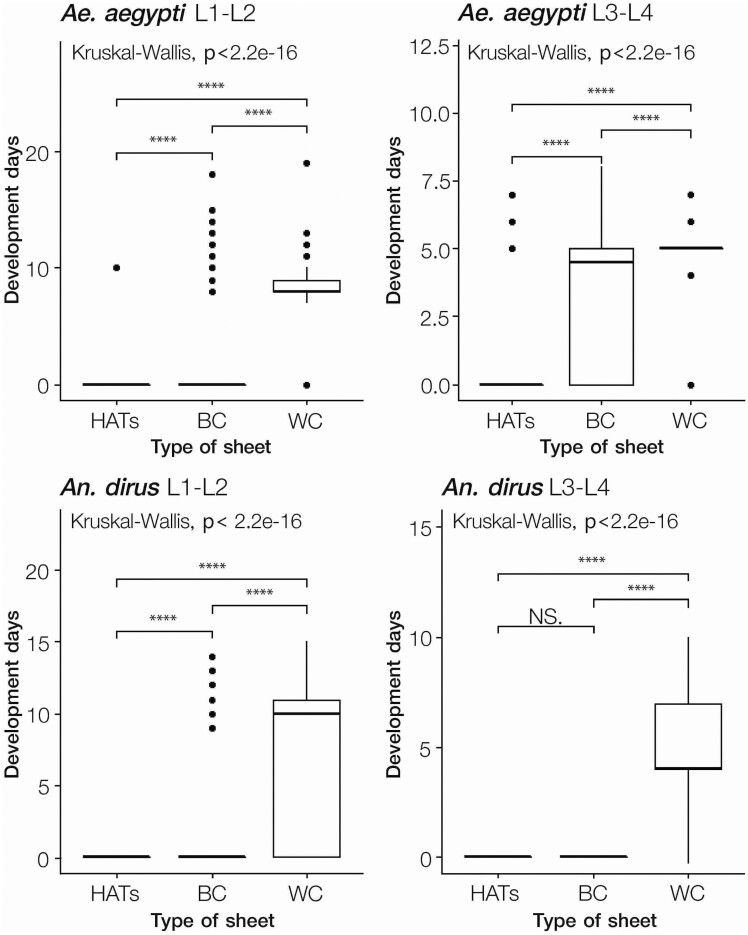
Development duration of surviving mosquitoes from larvae to adult. Time of development (d) from surviving larvae to adult stage *Ae. aegypti* (top) and *An. dirus* (bottom) when exposed to HATS, BC, or WC sheets as early- (left) or late-stage larvae (right). ****P < 0.0001; NS, not significant, analyzed by Kruskal–Wallis test. Abbreviations: Refer to Fig. 1 on HATS, BC, and WC; RO, reverse osmosis water.

The days taken to develop from early-stage larvae (L1–L2) into adults of the surviving *Ae. aegypti* were significantly different between the types of sheets (Kruskal–Wallis, *H* = 559.52, *p* < 0.0001). The late-stage larval (L3–L4) to adult development in the HATS group was also significantly different from the development days of the BC and control cup groups (Kruskal–Wallis, *H* = 341.30, *p* < 0.0001) (Fig. 4). Because no *An. dirus* early- or late-stage larvae survived in the HATS container, the lifecycle was not completed, and the development time could not be calculated. Moreover, the development time of early-stage *An. dirus* was significantly different between the BC and WC containers (Kruskal–Wallis, *H* = 472.178, *p* < 0.0001) (Fig. 4). Both were significantly different from that of the HATS container. *Anopheles dirus* late-stage larvae did not survive in the HATS and BC containers (not significant); however, that in both (HATS and BC) was significantly different from that in the WC container (Kruskal–Wallis, *H* = 767.244, *p* < 0.0001).

Altogether, exposure to HATS of the two mosquito species resulted in the death of almost all early-stage larvae (<1% survival rate). Furthermore, it had more effect on the survival rate of late-stage *An. dirus* than on that of *Ae. aegypti*. The survival rates of HATS-exposed larvae were significantly different from those of the BC and WC control larvae. However, it had a greater influence on the survival rate of late-stage *An. dirus* than on that of early-stage *An. dirus*. Early- and late-stage *An. dirus* larvae were both sensitive to the HATS compared to *Ae. aegypti*, of which only the early-stage larvae were sensitive. Subsequently, the HATS affected the early-stage more than the late-stage of *Ae. aegypti* larvae, which showed a low survival rate and increased time to adult emergence.

### Effect of HATS on Submersion of Adult Mosquitoes, Egg Hatching, and Number of Eggs Laid

#### Aedes aegypti

**Fig. 5.**
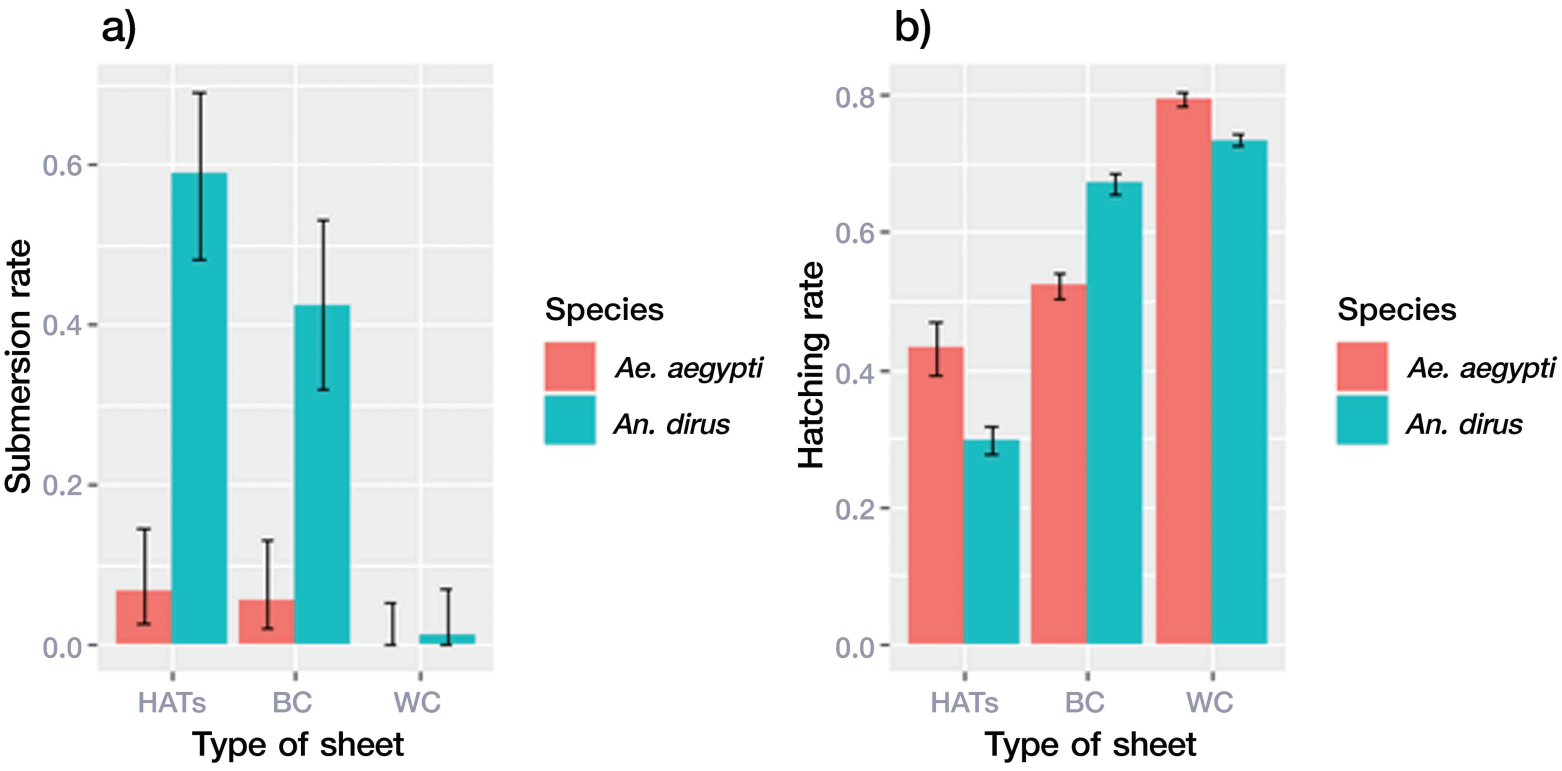
Submersion of female mosquitoes and egg hatching in three types of oviposition cups. Mean submersion rate of 30 engorged female mosquitoes (*Ae. aegypti* and *An. dirus*) in three types of oviposition cups in adult cages (30 × 30 × 40 cm) a) and the percentage hatching rate of laid eggs b) are represented in the graph with standard error bars. Abbreviations: Refer to Fig. 1 on HATS, BC, and WC; RO, reverse osmosis water.

The submersion of blood-fed females of *Ae. aegypti* submerged with HATS (6.67%) and BC sheets (5.56%) was found in the oviposition cups; however, a similar phenomenon was not observed in the WC control (Fig. 5a). The fewest eggs were laid in the HATS group (Table 1). Furthermore, the hatching rates (Fig. 5b) were significantly different (chi-square = 889.6, df = 2, *p* < 0.0001) among the eggs exposed to the HATS (43.11%), BC (52.32%), and WC control (79.35%) (Fig. 5b).

**Table 1.** Number of eggs laid by engorged female *Ae. aegypti* and *An. dirus* in oviposited cups

Species	*N*	HATS	BC	WC
*Ae. aegypti*	90	661	2,905	6,143
*An. dirus*	90	1,936	3,311	8,222

Abbreviations: HATS, hydroxyapatite-binding silver/titanium dioxide sheet; BC, blue sheet control; WC, white sheet control.

#### Anopheles dirus

The submersion rate of *An. dirus* females in water containing the HATS (58.89%) was significantly higher than that of the WC control (1.11%) with the white sheet (Fig. 5a), and the total number of eggs laid was different among the groups (Table 1). There was no difference in submersion, but there was a difference in hatching rates between the HATS and BC sheet (chi-square = 687.95, *p* < 0.0001). However, the 29.59% hatching rate of eggs oviposited in HATS was significantly different (chi-square = 1321.5, *p* < 0.0001) from that in the WC control (73.41%) sheet conditions (Fig. 5b).

## Discussion

The HATS is composed primarily of TiO_2_, silver, and apatite. Apatite can absorb bacteria and fungi; adding silver to TiO_2_ improves its catalytic efficacy and antibacterial activity by absorbing not only ultraviolet light (below 380 nm) but also visible light in the range of 700–800 nm (Durango-Giraldo et al. 2019, Okazaki et al. 2022). Similarly, apatite-TiO_2_-coated cotton fabric used in the study by Kangwansupamonkon et al. (2009) showed antibacterial activity against *Staphylococcus aureus*, *Escherichia coli*, and *Micrococcus luteus*. The apatite-coated TiO_2_ was irradiated and activated. It was proposed that the killing effect was mediated by ROS, such as OH^−^, O^2−^, and H_2_O, generated on the irradiated TiO_2_ surface, which may attack and decompose polyunsaturated phospholipids in bacteria (Maness et al. 1999). The ROS production may break down the cell wall and outer membrane, causing leakage of the cell content and resulting in cell irregularities and depressions (Kangwansupamonkon et al. 2009).

In this study, the hydroxyapatite-binding silver/titanium dioxide compound particle was used to treat nonwoven fabric sheets (HATS) at 0.1635 g of 0.0121 m^2^ sheet, along with a defoamer, and phthalocyanine blue color. The compound-treated sheet (HATS; developed by DR.C Medical Medicine Co., Ltd. in Tokyo, Japan; https://drciyaku.jp/mechanism.html) was lethal to larval mosquitoes. The amount used may be sufficient to kill the larvae of *Ae. aegypti* and *An. dirus* laboratory strains; however, questions remain regarding its applicability in the field. Not only did 0.1635 g of the compound kill larval *Ae. aegypti* and *An. dirus*, but the defoamer and phthalocyanine blue color also destroyed the larvae, as the larvae maintained with the BC (noncompound-treated sheet) showed a lower survival rate. The BC sheet also affected early-stage larvae and had a marked effect on late-stage *An. dirus*. Both the HATS and BC sheets contained defoamer and blue phthalocyanine color, whereas the WC sheet did not. We have no explanations for these phenomena; however, these types of chemicals may cause larval mortality owing to synergistic effects. Although BC could kill the larvae, it was less effective than HATS, which killed all larvae. The important thing regarding BC and HATS was the difference in hatching ability; this was especially noted in *An. dirus*.

There seemed to be a delay in the time to complete the lifecycle for the larvae of both species that survived under HATS and BC conditions, although most larvae perished, especially those in the HATS containers. The submersion rate of *An. dirus* gravid females in water containing the HATS was higher than that of *Ae. aegypti* under the same conditions. This difference is likely attributed to the typical oviposition habits of each species. *Anopheles dirus* females lay eggs on the surface of the water, whereas *Ae. aegypti* females primarily lay eggs on the water’s edge. Similar to water striders, mosquitoes can usually float on the surface of the water. Since water striders float owing to surface tension, HATS may impair the surface tension of water. Finally, we noted that *An. dirus* was more sensitive to HATS than *Ae. aegypti*, resulting in lower egg hatching, lower survival, and higher submersion rates. Therefore, the killing effects of HATS appeared to be different for each mosquito species. The amount of HATS needed for a killing effect should be adjusted for each species. Furthermore, the hatching rate of eggs exposed to the HATS was significantly different from those of the WC control group for both *Ae. aegypti* and *An. dirus*.

The mechanisms by which HATS acts on *Ae. aegypti* and *An. dirus* are unclear. Silver and TiO_2_ have antibacterial effects (Tudu et al. 2020); however, the relationship between their antibacterial effects and lethal effects on mosquitoes is unknown. The compound may penetrate the intracellular space and then either bind to sulfur from proteins or phosphorus from DNA, which in turn leads to denaturation of organelles, enzymes, and finally reduced ATP synthesis, similar to the mechanism behind the effect of AgNPs on *Ae. aegypti* (Skorb et al. 2008, Sundaravadivelan et al. 2013).

Although it is unknown exactly how ROS produced by the hydroxyapatite-binding silver/titanium dioxide compound affect mosquito larvae, ROS caused membrane damage in *Ae. aegypti* larvae under conditions of oxidative stress (Costa et al. 2022) and low egg production and poor developmental in *Drosophila melanogaster* (Diptera: Drosophilidae) (Philbrook et al. 2011).

On the other hand, TiO_2_ can regulate a material’s surface’s wettability. The surface turns from hydrophobic to hydrophilic when exposed to UV light (Wang et al. 1997). The hydrophobic surface of mosquitoes makes them water resistant. However, HATS-treated water was shown to hydrophilically cling to the surface in our unpublished observation. Thus, we speculated that adult mosquitoes perish by being submerged into the water owing to the hydrophilization of their legs, which land on the surface of the HATS-treated water.

The surface of fresh mosquito eggs laid on plain water changes from milky white to black and forms hard shells that are impermeable to the water (Farnesi et al. 2015). However, whether TiO_2_ is involved in the formation of this shell is unknown. The surface of eggs laid in HATS-treated water turned black. However, infiltration of the treated water into the eggs may continue owing to the hydrophilic properties of TiO_2_.

The limitations of this study are that the half-maximal effective concentration (EC_50_) and the precise mechanism of the killing of effect of HATS were not elucidated, warranting further studies. EC_50_ is often used to evaluate mosquito mortality using juvenile hormone-like insecticides. When evaluating the lethal effect of HATS on mosquitoes, it is necessary to consider values such as the number of adults submerged in water, the number of unhatched larvae, and the number of abnormally emerged adults.

In conclusion, we demonstrated that HATS is a potential new mosquito control measure, especially against *Ae. aegypti*, when it is attached to the water container. It prevented mosquitoes to lay eggs by deterring oviposition, resulting in fewer eggs laid (approximately 10% from their capacity) in the container, with a lower hatching rate (41%), and a very low proportion of larvae reaching adulthood (<1%). Although the laboratory results for *An. dirus* were excellent, its application in the field may be limited by the difficulty of attaching HATS in breeding sites, such as river streams. However, HATS has numerous advantages. First, it is safe to humans as particle sizes >100 nm cannot penetrate human skin (Schneider et al. 2009). Second, it can affect all stages of mosquitoes owing to its ovicidal, larvicidal, and repellent effects, as well as submersion of gravid females when laying eggs. Third, it has no smell, unlike commonly used chemicals in vector control programs, such as temephos, which has a strong smell, and thus most people dislike using it (Phuanukoonnon et al. 2006). Thus, future studies should formulate hydroxyapatite-binding silver/titanium dioxide particles in the form of granules or tablets, which are easy to apply in the container. The effective dose, longevity, and resistance of mosquitoes against the compound require further investigation.
